# Applying Social Network Analysis to Understand the Knowledge Sharing Behaviour of Practitioners in a Clinical Online Discussion Forum

**DOI:** 10.2196/jmir.1982

**Published:** 2012-12-04

**Authors:** Samuel Alan Stewart, Syed Sibte Raza Abidi

**Affiliations:** ^1^NICHE Research GroupFaculty of Computer ScienceDalhousie UniversityHalifax, NSCanada

**Keywords:** Web 2.0, health knowledge, attitudes, practice, knowledge management, information dissemination, pain, pediatrics, pediatric hospitals, education, professional, electronic mail

## Abstract

**Background:**

Knowledge Translation (KT) plays a vital role in the modern health care community, facilitating the incorporation of new evidence into practice. Web 2.0 tools provide a useful mechanism for establishing an online KT environment in which health practitioners share their practice-related knowledge and experiences with an online community of practice. We have implemented a Web 2.0 based KT environment—an online discussion forum—for pediatric pain practitioners across seven different hospitals in Thailand. The online discussion forum enabled the pediatric pain practitioners to share and translate their experiential knowledge to help improve the management of pediatric pain in hospitals.

**Objective:**

The goal of this research is to investigate the knowledge sharing dynamics of a community of practice through an online discussion forum. We evaluated the communication patterns of the community members using statistical and social network analysis methods in order to better understand how the online community engages to share experiential knowledge.

**Methods:**

Statistical analyses and visualizations provide a broad overview of the communication patterns within the discussion forum. Social network analysis provides the tools to delve deeper into the social network, identifying the most active members of the community, reporting the overall health of the social network, isolating the potential core members of the social network, and exploring the inter-group relationships that exist across institutions and professions.

**Results:**

The statistical analyses revealed a network dominated by a single institution and a single profession, and found a varied relationship between reading and posting content to the discussion forum. The social network analysis discovered a healthy network with strong communication patterns, while identifying which users are at the center of the community in terms of facilitating communication. The group-level analysis suggests that there is strong interprofessional and interregional communication, but a dearth of non-nurse participants has been identified as a shortcoming.

**Conclusions:**

The results of the analysis suggest that the discussion forum is active and healthy, and that, though few, the interprofessional and interinstitutional ties are strong.

## Introduction

### Objectives

The provision of quality patient care necessitates that health practitioners be informed about the best evidence concerning clinical diagnostic and therapeutic strategies, and more importantly be able to translate this knowledge into their clinical practices. Research has demonstrated, however, that vital medical research is often underutilized in clinical practices [1-3], resulting in suboptimal care. Studies have shown that 30-40% of patients do not receive treatment supported by evidence-based knowledge, and up to 25% receive unnecessary or potentially harmful care [4,5]. It is important, therefore, to develop innovative mechanisms that can help to effectively translate explicit knowledge into clinical practice to improve patient care.

Knowledge translation (KT) entails the implementation and enactment of knowledge dissemination strategies to effectuate the rapid uptake of new health knowledge into clinical practice [6]. Traditional KT strategies—including face-to-face sessions, workshops, oral presentations, and published media—have been successfully applied to translate new findings, methods, and policies into practice. Pursuing KT as a collaborative exercise can encourage peer-driven growth, an essential component of a community of practice [7]. A community of practice comprises a group of people that share a common interest but differ in knowledge and experience, and are interested in interacting with each other in order to share and advance their knowledge and the subject area. KT in a community of practice, therefore, can be perceived as the sharing of best evidence, contextualizing that evidence with personal experiences and observations, and operationalizing the evidence via practical situation-specific strategies and recommendations.

The emergence of Web 2.0 tools offer opportunities to pursue innovative approaches for health KT [8-10]. Web 2.0 tools, such as discussion forums, blogs, and mailing lists, provide an alternative to face-to-face knowledge dissemination activities by offering a virtual KT environment where community members from different geographical locations, different professional backgrounds, and different expertise levels can congregate and collaborate to disseminate explicit knowledge and influence practice change [7]. In practice, an online discussion forum engages participants in an asynchronous KT dialogue through which not only the intended explicit knowledge is disseminated, but also experiential knowledge - the professional experiences, insights, and observations of what worked and what did not work in specific clinical scenarios - can be shared in relation to this explicit knowledge. This contextualization of the explicit knowledge assists the KT exercise by allowing participants to see how the published knowledge can be applied to their clinical context. Notwithstanding the benefits of direct face-to-face KT strategies, Web 2.0 based KT methods can establish an active community of practitioners that interact with each other to share and translate knowledge into practice.

In this paper, we discuss a Web 2.0 KT environment targeting knowledge sharing within a community of practitioners interested in improving pediatric pain management. The Thai Pediatric Pain Discussion Forum was developed to facilitate knowledge sharing between an online community of practitioners around the topic of pediatric pain management [11]. This KT intervention was part of a broader global health project, conducted in collaboration with Canadian and Thai research teams, that aims to improve the awareness of pain in children and to reduce the knowledge gaps in pediatric pain management in 7 different hospitals in Thailand [11]. The objectives of the project were to elevate awareness of pediatric pain amongst health practitioners, standardize pediatric pain management across hospitals, share knowledge between practitioners to reduce knowledge gaps, and improve practices about pediatric pain management. The discussion forum was designed as a KT tool, intended to engage practitioners from different hospitals and professions to foster a pediatric pain community of practice.

The online discussion forum has been active for over 3 years and has provided a viable medium for pediatric care professionals to instigate a number of topic-specific discussions to share both their experiential knowledge and explicit knowledge resources (such as guidelines, research articles, presentations, etc) with the intent to collaboratively reduce the knowledge gaps that exist with regards to pediatric pain management. The knowledge sharing process generally proceeds as follows: (1) a practitioner seeks a solution or advice to a problem by presenting it to the discussion forum, (2) members of the community with interest and expertise related to the problem respond and moderate the discussion, (3) an online dialogue ensues in which practitioners highlight best evidence, shared experiences, and related theory to help address the question posed by a community member, (4) the knowledge shared in the discussion is disseminated via the discussion forum to the entire community of practitioners. An important aspect of the pediatric pain discussion forum is that the discussions – manifested as a series of messages on a specific subject, referred to as a “thread” – are archived and can be analyzed for both the knowledge content of the discussions and also for understanding the KT patterns between the community of practitioners.

To understand the dynamics of the knowledge sharing with the pediatric pain community, we investigated the following aspects of knowledge sharing:

1. What are the participation behaviors across different hospitals and different occupations? Are there dominant institutions or professions within the community?
2. What is the relationship between reading and posting within the forum? Are there members of the discussion forum that are active in one but not the other?
3. Who are the most active and most influential members of the community?
4. Can a central group of active members be identified and differentiated from the rest of the community?
5. Is there strong interaction between members from different occupations and/or different hospitals?

The first 2 questions were answered using statistical analysis and visualizations, while the last 3 were addressed using Social Network Analysis (SNA). We investigate the research questions above through our analytical tools and provide a quantitative measurement, in terms of social networks and communication patterns noted within the online community of practice. We conclude with a discussion on the utility of Web 2.0 tools for KT, particularly in the context of knowledge sharing within special interest online communities.

### Background

The use of Internet to facilitate social interaction and KT is a well-studied area. Wellman and colleagues [12] surveyed a large number of Internet-based communities to investigate how the principles of online communities could be used in workplace interactions. They explored primitive communication tools such as email, list servers, and usenet groups to establish how these tools could improve communication by bridging physical boundaries. In a series of papers [13-16], Wellman and others extended this analysis to explore online communities as social networks and, using SNA, presented analytical methods to develop a better understanding of how people communicate in an online environment. Using SNA as a tool for understanding online communities forms the basis of our research project to observe and understand the communication patterns in the pediatric pain discussion forum.

The Web has become a choice medium for discussion forums and online communities around health. Eysenbach and colleagues [17] found 24,000 health-related discussion groups within Yahoo groups alone (in 2004). The authors attempted to review the efficacy of discussion forums as a medical intervention, but found a dearth of quality papers. They found 45 papers representing 38 studies, of which only 6 studied internet-based interventions as the primary focus of the project. One of the conclusions drawn by the authors was that there is no robust evidence that online communities impacted health outcomes, but that there are clear health benefits when seeking information from online communities. It was noted that as the number and size of virtual health communities increases, it is vital to understand the implications of these communities on health attitudes, knowledge, and outcomes. One of the objectives of our project is to outline how SNA can be used as a tool to understand online communities, so that their efficacy as an intervention may be more properly addressed.

Virtual communities succeed when there is an “intrinsic desire” to communicate and share health knowledge and experiences within the community [17]. This finding is confirmed in more recent experiences of using discussion forums to facilitate education and KT. For example, in a study where students in an anatomy class had 8% of their grade linked to their participation in a discussion forum, 83% of the students found the boards useful in improving their team building and critical analysis skills [18]. This finding was replicated by Kuhn et al [19] who found that a moderated pretest discussion forum as a tool for facilitating communication between nursing students significantly improved students' grades. Valaitis et al [20] designed a discussion forum to facilitate the establishment of a virtual community of practice for Community Health Nurses. For a dispersed community with a dearth of quality knowledge [20], a discussion forum provided a key KT tool for the participants, providing them with a way to connect to their peers. The authors noted that “the development of effective Communities of Practice is dependent upon the ability of individuals in the community to critically interpret, respond, and share information with colleagues” [20].

In contrast, when participation is neither required (via grades) nor requested by the community, it has been noted that participation tends to wane. In a study comparing online journal clubs to face-to-face journal clubs, researchers found a huge gap in participation rates, with lower participation in Internet journal clubs. [21]. Though the authors stated that journal clubs were mandatory, there was no punishment for not participating. With no explicit inducement to participate and no intrinsic desire from the residents, the discussion forum faltered. Therefore, we argue that it is important to not just provide another communication tool to practitioners, but to build a community of practice [22] within which people can communicate with their peers and share information such that the entire community benefits from the insights led by a few individuals.

Communities of practice [22] are defined as a group of people who share a concern or a passion for something they do, who interact regularly to learn how to improve. A community of practice has 3 dimensions: the domain, the community, and the practice [7]. The domain is the area of interest defined by the group. The community is the individuals with a common interest to learn from one another. These individuals do not have to work together on a daily basis, nor do they have to meet face-to-face. The defining quality of the community is that the individuals interact to learn from each other***.*** The practice is what the community members are striving to improve, taking the knowledge they glean from the community and putting it to use in their everyday activities.

To establish a viable online community of practice, it is important to take a methodical approach for both the development and operation of the online KT environment. One such approach is presented by the Leveraging Internet for Knowledge Sharing (LINKS) model, which presents a conceptual framework to help establish online communities of practice for specialized knowledge sharing using Web 2.0 tools [10]. The LINKS model identifies the key determinants of an online knowledge sharing environment in order to systematically conceptualize and implement a purposeful health knowledge sharing environment for an online community of practice. The LINKS model characterizes healthcare knowledge sharing solutions at 3 interrelated levels: concepts, operations, and compliance. The conceptual level stratifies knowledge sharing into 3 dimensions: the knowledge modality, the knowledge sharing context, and the knowledge sharing medium. The operational level addresses technical infrastructure issues pertaining to establishing a culture of collaboration between the stakeholders. The compliance level addresses the underlying issue of perceived trust in the system. For this project, we used the LINKS model to guide the development and operation of the Pediatric Pain Discussion Forum. A more detailed explanation of the implementation process for the discussion forum is described in the original paper [11].

## Methods

The objective of this project is to evaluate the communication patterns within an online community of practice. The archives of the discussion forum from April 2009 until June 2011 were used to evaluate the usage of the discussion forum and to study the communication patterns observed within the community.

Simple statistical summaries are used to provide a broad overview of the members and their participation in the social network. Visualizations of the communication patterns within the social network can provide insights into the participation behaviors of different hospitals and different professions (question 1), and the relationships between reading and posting on discussion threads (question 2). Because of the extremely non-normal distribution of posts and reads per person, Kruskal-Wallis tests were used to investigate whether people from specific institutions or specific occupations tend to post or read more. SNA was used to delve deeper into the underlying network structure of the discussion forum. Below we provide a detailed account of the SNA methods and results.

Social Network Analysis (SNA) builds on the principles of graph theory to study the relations between actors, and how they influence the overall network. SNA represents communication in terms of *nodes* (which represent actors/members), and *edges* (which represent communication ties) [23,24]. Whereas traditional statistical analysis focuses on actors and their personal attributes, SNA focuses on the relations between actors, and not the actors themselves.

Discussion forums can be represented as 2-mode networks, in which there are 2 classes of nodes, and the edges go from one class to another (see Figure 2 for the 2-mode and 1-mode networks). For this project the 2 classes of nodes are: (1) the discussion forum members, and (2) the threads they communicate on, and an edge indicates that a specific member has communicated on a specific thread. Representing the data as a 2-mode network allows the threads to be viewed as a KT event that community members participated in. Because many SNA methods are designed for 1-mode networks, a transformation of the 2-mode network is sometimes necessary. An undirected 1-mode network is created from the discussion forum members, in which a tie between 2 members indicates they have communicated on a thread, and the value of the tie is the number of threads they have both communicated on. Note that a 1-mode thread network could also be created, but that was not used for this project, as it did not provide any meaningful insight into the network.

Centrality measures can be used to identify the most active and influential members of the community (question 3). They can provide insight into the most important actors in the social network; those that are at the center of the network in terms of communication between individuals. 3 different centrality measures will be used: (1) degree centrality measures the number of ties an individual node has, (2) closeness centrality measures how quickly a single node can reach all other nodes, and (3) betweenness centrality deems a node central to the network if they are often used as a path between 2 other actors. All 3 measures are normalized to a (0,1) scale for simpler interpretation (see Wasserman [23] and Hanneman [24] for the technical calculations of these values, and Borgatti et al [25] for adaptations to 2-mode networks).

Finding a central group of community members (question 4) can be done using core-periphery analysis. Core-periphery analysis is a clustering algorithm that assumes that there is a core set of nodes at the center of the social network, and a periphery set of nodes that connect to that core [25,26]. It will be used to identify the members and threads that are at the center of the 1- and 2-mode networks. For the 1-mode member network, a measure of “coreness” can be calculated. Coreness is the measure of how central that member is to the network and can be thought of as another measure of centrality.

Finally, the interaction between different hospitals and professions (question 5) needs to be studied using group-level centrality measures [27]. For this analysis, group-level measures were calculated across both occupation and hospitals to explore the intergroup communication patterns. The same 3 individual level centralities can be calculated across groups: Group degree measures the number of connections from within a group to members outside it, group betweenness measures the proportion of shortest paths between 2 non-group members that pass through the group, and group closeness measures how close the group is to all other members in terms of direct ties and paths. The purpose of group-level analysis is to determine whether there are groups of members (either of a certain profession or from a certain institution) that are influencing the flow of information through the social network. The plots of reads and posts in Figure 4 demonstrate that nurses and members from Sringagrind hospital are the most active in terms of participation, but group-level measures will provide insight into whether these or other groups are facilitating more communication or are at the center of the community.

## Results

### Data

The data for this project were extracted from the forum in June 2011, and represent the communications from the initiation of the discussion forum, April 1, 2009, up to June 30, 2011. The data were analyzed using the statnet library in R version 2.12.2 [28] and UCINET [29].

**Figure 1:**
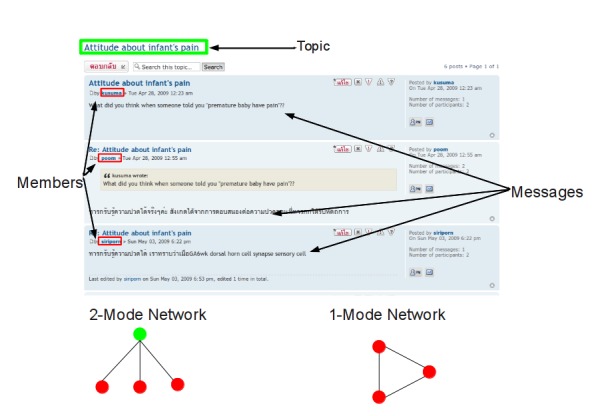
An example thread from the discussion forum, identifying how the threads are presented, where the data is extracted from, and how the 1- and 2-mode networks are created.

For every post on the discussion forum, the database records the member that made the post, the thread in which the post was made, and the time the post was made. The system also records the most recent time that a member has read a specific thread. Figure 1 contains an example of how the data is presented in the forum, and the origin for the information used in the study.

**Figure 2:**
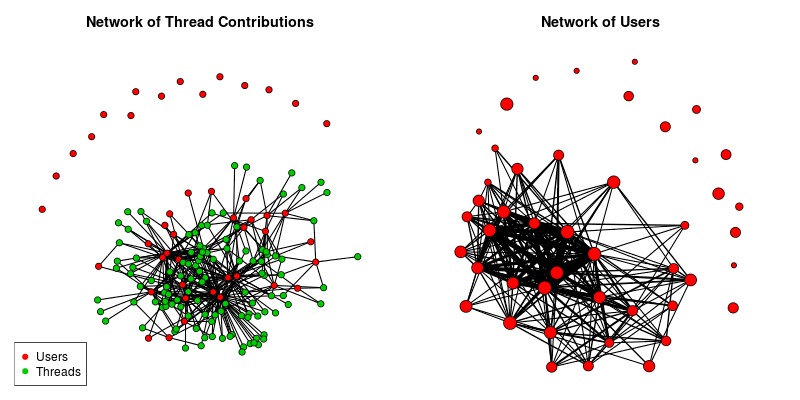
Plots of the 2-mode network (left), and member network (right). For the member network the width of an edge is determined by the edge value, and the size of the member node represents the number of threads that the user has read.

The discussion forum has 46 unique members, of whom 31 have posted at least once. There were 568 posts to the discussion forum on 115 threads, resulting in an average thread length of 4.94 (range of 1-25 posts per thread, median of 3). Of the 31 active posting members, 12 have posted to 10 or more threads, and 23 have read 10 or more threads. Figure 2 presents the 2 social networks being studied.

There were 21 threads (18%) that did not receive a response in the discussion forum. These threads averaged 6.8 reads per thread (range of 2-12 reads, median of 7), with all receiving at least one and all but one receiving more than 3. Not all threads are expected to receive a response: many of them are conference or workshop announcements, or informing the community that a new resource has been added to the resource center. Without more insight into the content of the thread it is difficult to determine if the thread was left unanswered because the question was difficult, the community was uninterested, or it was simply an announcement. For a community to successfully form, it is imperative that questions be answered in a timely manner in order to encourage participation. With only 18% of the threads being isolates, and many of those being expected, the isolate rate on the forum is acceptable, but efforts should always be made to ensure that threads are responded to in a timely manner.

**Figure 3:**
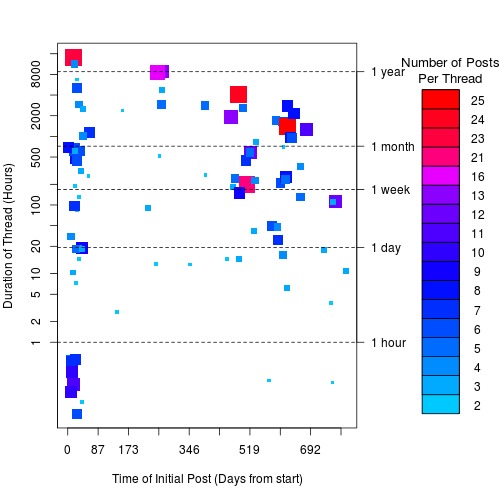
A plot detailing the number of posts and duration of threads as a function of time in the forum. The x-axis represents the date of the first post in the thread, the y-axis represents the time between the first and last post (note that the y-axis is in a log-scale). Each square represents a thread, and the size and colour of the square are defined by the number of posts in the thread.

To get a better understanding of the persistence of a thread/topic—the duration over which the thread is relevant to the community—we used the timestamps of the messages that were posted to a thread (the software does not capture the read times in the system, therefore we cannot determine thread persistence based on it being read). Figure 3 presents the number of posts per thread and the number of hours between the first and last post to the thread. Note that the threads that did not receive a response are omitted from this graph (as they only have 1 timestamp, and thus have a duration of 0).

Table 1 shows some interesting characteristics of their discussion forum with regards to thread activity. For instance, 52 threads were active for a relatively long period spanning more than a week, whereas there were 10 threads that were active only for an hour but in this short time period the activity level (in terms of number of posts) was extremely high. We argue that such short-duration threads could have been practice-related questions that received a rapid response from a few active community members, whereas the more persistent threads could have been discussions that did not relate to critical patient care, resulting in a more drawn out and in-depth discussion. Without a content analysis it is difficult to investigate this phenomenon any further, but the thread durations do suggest a variety of knowledge sharing characteristics.

**Table 1:** The number of posts per thread for each of the thread duration categories.

	Number of threads	Mean number of posts	Min	First quartile	Median	Third quartile	Max
≤ *1 hour*	10	6.2	2	2.75	6.5	9.25	11
≤ *1 day*	18	3.1	2	2	3	3	9
≤ *1 week*	14	4.9	2.	3	4	6.75	12
≤ *1 month*	25	5.6	2	3	5	6	21
≤ *1 year*	24	7.5	2	3.75	5.5	8	25
*> 1 year*	3	13.3	4	8.5	13	18	23

### Understanding Participation Across Institutions and Occupations

**Figure 4:**
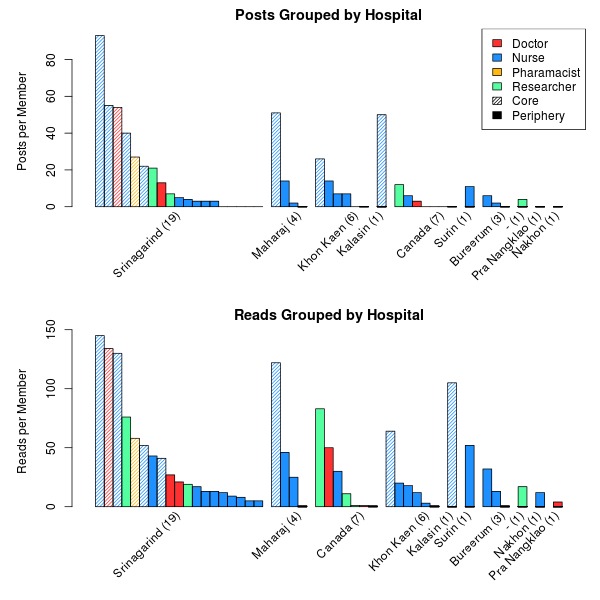
The number of posts and reads, grouped by hospital, with the number of community members in each group denoted within the brackets. Each bar within a hospital represents an individual member. The colour of the bar is an indication of their occupation and the shading on the bar indicates if they are a member of the core or not (see Table 6).

In the first question we wanted to understand the participation behavior of community members belonging to different institutions, as this will inform us about the propensity for a knowledge sharing culture within the institution. Participation in the online discussions was measured based on reading and posting activities performed by each community member. Figure 4 presents the number of posts and reads from the discussion forum, stratified by institution, with the colour of the bar identifying the profession of the community member. The results show that the hospital in Srinagarind is by far the most active, accounting for 62% of the posts and 51% of the reads across the discussion forum. Though there are more community members from Srinagarind there is no evidence to suggest that members from Srinagarind are either posting or reading more individually. Kruskal-Wallis tests resulted in *P* values of 0.51 and 0.56, so there is no evidence to suggest that there is a difference in posting or reading rates per person across institutions.

Next, we analyzed the participation behaviors across different professions in order to understand the propensity for knowledge sharing and translation from an occupational standpoint. The colours of the bars in Figure 4 indicate the profession of each member. The Figure demonstrates that nurses are the most active professionals involved in the discussion forum–nurses accounted for 77% of the posts and 67% of the reads, with one doctor and one pharmacist significantly contributing to the discussion forum. A Kruskal-Wallis test was again performed to study whether there was a difference in posting or reading rates per person between occupations. *P* values of 0.54 and 0.73 for posting and reading respectively demonstrate that there is no evidence that people from different occupations post or consume content from the discussion forum at different rates.

Looking at the interaction of institution and occupation, we observe that the majority of the smaller hospitals only engaged nurses in the project: of the 5 doctors in the community, 3 are from Srinagarind and one is Canadian, so only one Thai doctor outside the major research center engaged in the community. As well, the only active pharmacist is from the most active hospital.

### Comparison of Reading and Posting on the Discussion Forum

**Figure 5:**
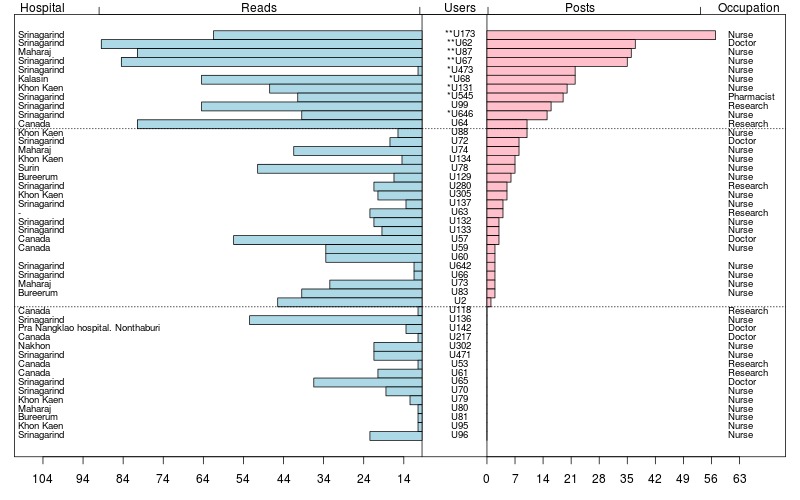
Comparing reads and posts for individual members (note that the partitioning of the figure into 3 sections is done arbitrarily). For this figure, posting is not counted as reading, so the reads represent a member reading a thread and not commenting on it. Members with * by their name are from the 1-mode core (Table 6), and members with ** by their name are from the 2-mode core (Table 5).

For KT purposes, it is important that practitioners engage with the online discussions even if it is to the extent of reading and then internalizing the experiential knowledge shared by other practitioners. Figure 5 presents the number of posts compared to the number of reads per member, ordered by number of posts. It was noted that those members who post a lot also read a lot, representing a potential group of “super-users” that are active in many threads within the community. The members in the middle section of the Figure have low posting numbers but high read numbers; these members were regarded as active in the discussion forum, with a tendency to contribute selectively but read a broad spectrum of topics. The final group are those members that have not posted but have read threads, and we have noted that there are several members that are participating in the network by reading threads but not contributing to them. This is not a negative outcome, as the intent of the discussion forum is to share knowledge across a community, so it was anticipated that some community members might not be in a position to contribute for various reasons.

### Member and Network Centrality

Centrality measures can provide information about the activity levels of the individual members, along with the overall activity status of the social network. Based on the online discussion data, we developed two 2-mode social networks, one for posting and one for reading. We then calculated standard centrality measures to identify the most active (or central) community members. Closeness, betweenness, post degree, and coreness are all from the post network, while read degree is from the read network.

**Figure 6:**
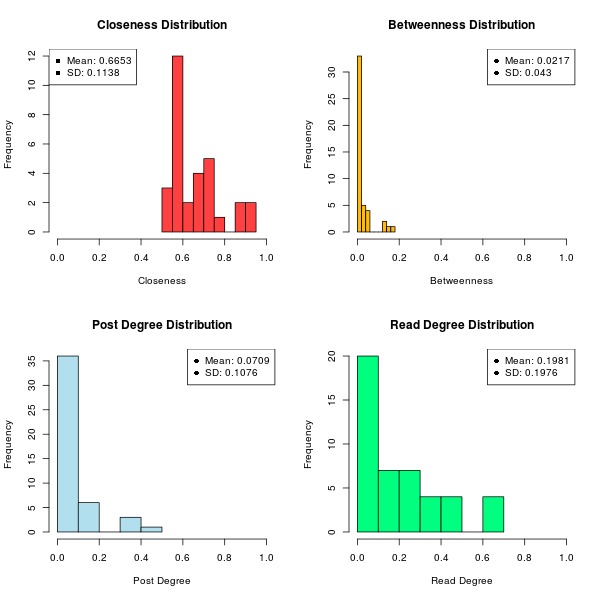
The distribution and summary statistics for the centrality measures.

Table 2 lists the centrality measures for the discussion forum for each member, and Figure 6 presents the distribution and summary statistics (mean and standard deviation) for each of the measures.

**Table 2:** A sample of centrality for social network members, ordered by coreness.

User	Closeness^c^	Betweenness	Post degree	Read degree	Coreness^c^
U173^ab^	0.88235	0.17551	57	52	0.47111
U62^ab^	0.90909	0.1281	37	80	0.43945
U87^ab^	0.90909	0.13642	36	71	0.40202
U68^b^	0.73171	0.02931	22	55	0.30356
U67^ab^	0.88235	0.15055	35	75	0.29276
U473^b^	0.76923	0.05866	22	1	0.25582
U646^b^	0.73171	0.03615	15	30	0.20707
U545^b^	0.69767	0.01607	19	31	0.20382
U131^b^	0.75	0.05669	20	38	0.17414
U99	0.75	0.05245	16	55	0.16883
U64	0.71429	0.04947	10	71	0.08668
U88	0.68182	0.02582	10	6	0.07642
U280	0.6	0	5	12	0.06166
U134	0.66667	0.0203	7	5	0.05432
U305	0.56604	0	5	11	0.04753
U73	0.625	0.00123	2	23	0.04276
U78	0.68182	0.02474	7	41	0.04188
U74	0.6	0.01207	8	32	0.04162
U60	0.58824	0	2	24	0.04099
U66	0.6	0	2	2	0.03835
U83	0.58824	0	2	30	0.03577
U57	0.6	0.00477	3	47	0.03419
U63	0.58824	0.0012	4	13	0.02502
U132	0.6	0.00231	3	12	0.02448
U72	0.61224	0.01586	8	8	0.02405
U129	0.58824	0.00131	6	7	0.02199
U133	0.55556	0	3	10	0.01979
U2	0.57692	0	1	36	0.01928
U137	0.53571	0	4	4	0.01398
U642	0.52632	0	2	2	0.01176
U59	0.51724	0.00099	2	24	0.0105
U53		0	0	1	
U61		0	0	11	
U65		0	0	27	
U70		0	0	9	
U79		0	0	3	
U80		0	0	1	
U81		0	0	1	
U95		0	0	1	
U96		0	0	13	
U118		0	0	1	
U136		0	0	43	
U142		0	0	4	
U217		0	0	1	
U302		0	0	12	
U471		0	0	12	

^a^ These users were members of the core of the 2-mode network

^b^ These users were members of the core of the 1-mode network

^c^ Isolate actors had to be dropped for these metrics

The max normalized degree in the network is 0.496, indicating that one member has communicated on nearly 50% of the threads. Looking at the posting histogram in Figure 6, however, the majority of the post degrees are below 0.2 and the mean is .07, indicating that members are not all contributing to all the threads. This is a positive finding, as a social network in which a single member or set of members have very high degrees means that a single member or set of members is dominating the social network. The read distributions are also encouraging. A mean of 0.20 means that the average member is reading around 20% of the content on the discussion forum, which represents around 23 threads.

The closeness results are high, which is a positive finding, and an expected one in a network with high degree values. A max closeness of 0.91 indicates that there are 2 members who have communicated directly with 91% of the other members on a single thread. With a mean closeness of 0.665 and a minimum closeness of 0.517, there is strong evidence to suggest that the community is well connected, and members can quickly connect to all other members of the social network via their shared thread communications. The isolates in the network are not included in the closeness calculations, but their disconnectedness is an issue that needs to be addressed.

Finally, the betweenness measures are quite low, which is a positive result, particularly given the high degree values. A high betweenness value indicates that that member acts as a communication gateway for the social network, and with very low betweenness values (a mean of 0.02 and a max value of 0.175) there is little evidence to support the idea that there are members acting as information conduits for the social network. This means that members of the community have multiple avenues to connect to their peers, which encourages more communication and knowledge sharing opportunities.

### Identifying a Core Group of Users

Core-periphery analysis helps identify the community members that are at the center of the network in terms of posting. The analysis was performed on both the 1-mode and 2-mode networks to try and identify which members and threads are at the center or edges of the network. Core-Periphery analysis on the 1-mode network identifies those actors that have strong connections with many other members, while analysis on the 2-mode network identifies the most central threads, and the actors that are associated with them. Once again, the isolate actors are dropped from the 1-mode analysis as their coreness cannot be calculated.

Table 2 presents the coreness of the members in the 1-mode network along with membership in the core for the 1- and 2-mode networks. The histograms in Figure 4 demonstrate where the core members fall in terms of occupation and hospital, and Multimedia Appendices 1 and 2 presents the 1-mode and 2-mode networks as matrices with the core and periphery clearly identified. The image matrices (denoting the average tie density of each of the sections of the network) are available in Tables 3 and 4 for the 1- and 2-mode networks respectively.

For the 2-mode network, the core members communicate, on average, in 43% of the core threads, and 27% of the periphery threads. The periphery actors, meanwhile, contribute to only 8% of the core threads and 5% of the periphery. For the 1-mode network, the core actors share 8.6 threads, and 1.3 threads with the periphery actors, who only share 0.277 threads with each other.

**Table 3:** Communication densities stratified by the core-periphery structure in the 2-mode network.

2-Mode network	Core	Periphery
Core (4 members; 27 threads)	0.438	0.273
Periphery (27 members; 55 threads)	0.083	0.051

**Table 4:** Communication densities stratified by the core-periphery structure in the 1-mode network.

1-Mode network	Core	Periphery
Core (9 members)	8.593	1.308
Periphery (22 members)	1.308	0.277

These results confirm the findings from previous sections, about the presence of a core-group of “super-users” that seem to generate the bulk of the content within the community.

### Calculating Group Centrality Indicators

The objective of the group centrality analysis is to explore the interactions between group members in order to determine how different types of professionals (such as nurses and doctors), or professionals from different institutions, interact as a community. The analysis is important to understand whether the online discussion forum managed to break the professional or institutional barriers that are typically prevalent in a healthcare system. We calculated group-level centrality indicators across both occupations and institutions, presented in Tables 7 and 8 respectively. Note that, since we are not considering read statistics in this analysis, we removed the disconnected members, as they would not contribute to the calculations of the group indices.

There are 215 ties in the 1-mode network representing ties between individual users, and the value of those ties represents the number of threads those 2 users have shared. The total value of the ties in the network is 674, with 431 (64%) being between different hospitals and 340 (50%) being between occupations. These values suggest that there is significant communication between occupations and between hospitals, but group centrality analysis can provide more insight into the relations. Tables 5 and 6 contain the communication counts between occupations and between hospitals respectively. The diagonal terms in these tables represents the intra-occupation communications, and the off-diagonal terms represent the interoccupational communications.

**Table 5:** The interoccupation communications.

	Doctor	Nurse	Pharmacist	Research
Doctor	4^a^ (0.0229)^b^	149 (0.851)	7 (0.04)	15 (0.0857)
Nurse	149 (0.242)	316 (0.513)	54 (0.0877)	97 (0.157)
Pharmacist	7 (0.101)	54 (0.783)	0 (0)	8 (0.116)
Research	15 (0.122)	97 (0.789)	8 (0.065)	3 (0.0244)

^a^The number of messages sent

^b^The proportion of total messages sent by that group

**Table 6:** The interhospital communications.

	no recorded hospital	Bureerum	Canada	Kalasin	Khon Kaen	Maharaj	Srinagarind	Surin
no recorded hospital	0 (0)	1 (0.0769)	0 (0)	0 (0)	6 (0.462)	1 (0.0769)	4 (0.308)	1 (0.0769)
Bureerum	1 (0.04)	0 (0)	0 (0)	2 (0.08)	4 (0.16)	2 (0.08)	14 (0.56)	2 (0.08)
Canada	0 (0)	0 (0)	1 (0.0196)	2 (0.0392)	4 (0.0784)	8 (0.157)	34 (0.667)	2 (0.0392)
Kalasin	0 (0)	1 (0.0105)	2 (0.0211)	0 (0)	5 (0.0526)	13 (0.137)	73 (0.768)	1 (0.0105)
Khon Kaen	3 (0.0236)	3 (0.0236)	3 (0.0236)	2 (0.0157)	12 (0.0945)	18 (0.142)	81 (0.638)	5 (0.0394)
Maharaj	1 (0.0073)	2 (0.0146)	5 (0.0365)	2 (0.0146)	7 (0.0511)	3 (0.0219)	115 (0.839)	2 (0.0146)
Srinagarind	3 (0.0090)	10 (0.0301)	18 (0.0542)	9 (0.0271)	29 (0.0873)	26 (0.0783)	226 (0.681)	11 (0.0331)
Surin	1 (0.0667)	1 (0.0667)	1 (0.0667)	1 (0.0667)	3 (0.2)	1 (0.0667)	7 (0.467)	0 (0)

For the occupations, as would be expected from the histogram in Figure 4, the nurses seem to dominate the social network. As a group they are connected to the rest of the network by a single step (see the normalized closeness of 1), and there is a nurse on 60% of the shortest paths between 2 members (Table 7). Looking at the other professions, the doctors and researchers are well connected, and the doctors fall on a number of shortest paths (13%), which is a promising result. The high closeness and betweenness scores indicate that there is interaction between professions.

It is difficult to interpret a number of the hospital results, as several hospitals are underrepresented, but Srinagarind is by far the most influential hospital, being completely connected to the other members in one step, and having 58% of the shortest paths go through them (Table 8). Also of note is the hospital at Maharaj, which is very well connected despite having only 3 active members. Once again, the high degree and betweenness measures are strong indicators that there is communication between hospitals.

**Table 7:** Occupation centrality indicators.

Group	n	Degree	Normalized degree	Closeness	Normalized closeness	Betweenness	Normalized betweenness
Nurse	22	9	1	9	1	0.6073	0.5398
Research	5	22	0.8462	30	0.8667	0.0852	0.082
Doctor	3	26	0.9286	30	0.9333	0.1301	0.1255
Pharmacist	1	17	0.5667	43	0.6977	0.0227	0.0219

**Table 8:** Hospital centrality indicators.

Group	n	Degree	Normalized degree	Closeness	Normalized closeness	Betweenness	Normalized betweenness
Srinagarind	14	17	1	17	1	0.5823	0.548
Khon Kaen	4	18	0.6667	36	0.75	0.1182	0.1138
Canada	4	18	0.6667	36	0.75	0.0296	0.0285
Maharaj	3	26	0.9286	30	0.9333	0.1369	0.132
Bureerum	2	15	0.5172	43	0.6744	0.0023	0.0022
-	1	9	0.3	51	0.5882	0.0021	0.002
Kalasin	1	19	0.6333	41	0.7317	0.0375	0.0363
Surin	1	16	0.5333	44	0.6818	0.0266	0.0257

## Discussion

This study investigated the dynamics of knowledge sharing through a Web 2.0 based medium - an online discussion forum - involving a specialized community of healthcare practitioners. Practice knowledge (also referred to as experiential knowledge) elicits peer-generated insights by health professionals about what worked, what did not work, and what to do in specific situations. There is a growing recognition that practice-related knowledge is a vital knowledge resource, supplementary to evidence-based resources, for health care practitioners who have to deal with complex and at times atypical clinical situations for which evidence-alone is at times not sufficient [30-32] Practice-related knowledge is not necessarily evidence-driven, yet it entails critical decisions, judgements, practices, and outcomes performed and observed by peer practitioners in specific clinical situations. We argue that both the experiential knowledge content and the associated mechanisms for its collection and translation to practice are of importance from a KT perspective. In this project, we examined the knowledge sharing dynamics in an online communication environment.

With the rapid adoption of social computing and mobile computing technologies, it is prudent to explore the application of new computing technologies to pursue new methodologies and methods for instituting KT programs. Web 2.0 based social interactions between like-minded practitioners offer new avenues for the creation and critique of experiential knowledge in an incremental and inclusive manner within a public space that is accessible to a wider audience where both the knowledge sharing medium and the inherent knowledge content serve as a KT resource. We believe that for KT, Web 2.0 based social computing technologies provide a ubiquitous and inclusive knowledge sharing method that can potentially overcome the geographical, temporal, social, and hierarchical barriers that challenge traditional KT methods [9,33,34] The efficacy of Web 2.0 tools for KT can be determined by analyzing the knowledge content being created and shared via an online discussion forum, whereas the effectiveness of Web 2.0 based KT programs can be gauged through the levels of user participation and knowledge sharing, which can be measured by analyzing the communication patterns between the online community of practice. In this project, we analyzed the communication dynamics of an online community of pediatric pain practitioners and our results not only explain the knowledge sharing patterns within the community of practice but can be generalized to serve as recommendations for developing a Web 2.0 based KT program.

In this study we posed 5 research questions to investigate the dynamics of knowledge sharing within a virtual community of practice. The objective of these research questions was 3 fold. First, to measure the participation rates of members belonging to different institutions and professions in order to understand whether certain institutions or professionals are more inclined to participate in online discussions. Second, to identify whether certain members have emerged as central figures to the various discussions, in order to identify and designate knowledge brokers/KT champions within individual institutions. Finally, to examine the degree of collaboration, in terms of knowledge sharing ties, which may have transpired across professionals from different institutions via the online discussion forum, since it allows geographically dispersed professionals to communicate and collaborate in a more ubiquitous manner. Our findings provide insights, leading to objectively derived recommendations, about the design of new models for KT, especially the use of Web 2.0 based collaboration technologies for KT across a virtual community of professionals.

We investigated the participation rates of the community members belonging to different professional groups and institutions. Based on both the post and read frequencies, it was noted that there is no difference between the participation rates of members belonging to different institutions, and that participation in the online discussions is driven more by an individual’s engagement with the online community rather than the member belonging to a specific institution. This is an interesting observation as it delineates participation level from the institution, suggesting members are self-motivated to participate in knowledge sharing as opposed to being influenced by their institution. It is worth noting that although the discussion forum has more members associated with a large urban hospital at Srinagarind, this did not mean that individuals from Srinagarind are more likely to contribute to the forum.

With regards to professional groups, our analysis shows a predominance of nurses being engaged in the discussions, though it should be noted that the project did engage a relatively large group of nurses for membership to the discussion forum. What is interesting to note is the lack of participation from the physicians—only one physician significantly contributed to the discussion forum. It is important to factor the influence of external motivational strategies geared to engage members to the online discussions. In the Thai project, each institution was assigned a nurse facilitator (ie, a KT champion) who was responsible for routinely encouraging pediatric professionals in his/her institution to participate in the knowledge sharing activities, including the online discussion forum. Indeed, the energy, expertise and enthusiasm of the individual nurse facilitators had an influence in the overall participation rates of institutions and professional groups. We did observe that some nurse facilitators were more successful than others in promoting the online discussion forum and engaging professionals to participate in online discussions. In moving forward with Web 2.0 interventions, it is important to ensure that the facilitators are willing and capable of engaging all potential users of the community, including those from outside their profession.

As both the medium of Web 2.0 based discussion forums and the method for knowledge sharing are new to some practitioners, there may be apprehension towards the use and utility of online discussion forums. We propose that to institute a vibrant Web 2.0 based KT program, it is prudent to implement certain member engagement strategies. One method is to promote the specialized online discussion forum as a knowledge resource by demonstrating the value of sharing/using experiential knowledge derived from peer practitioners. Another is to pursue active engagement and support of the members, especially in the initial stages of the online KT environment, to ease them into using the online discussion forum with ease and trust. We found that designated KT champions can both engage members and facilitate online discussions, which in the long run helped to maintain high-levels of participation, contribution, and KT. In our study, we notice that once members are properly engaged there was no significant difference between the participation rates across the different professions.

Social network analysis of the communication patterns of the entire community determined that the network is fairly well connected. High closeness scores indicate that members can readily connect to each other, and low betweenness scores suggest that the network does not depend on a single member for relaying information through the network. These are promising communication patterns, as they indicate that the community is not overly dependent on a single member or set of members to share knowledge, and that even disconnected members can readily connect through mutual friends. The centrality measures highlighted the presence of a set of highly active community members with high centrality rankings across all measures, and core-periphery analysis identified the same members as central to the community.

We investigated the relationship between reading versus posting at the online discussion forum. Our analysis identified 3 groups of users: actives posters, selective posters, and non-posting consumers (or lurkers) as indicated by the 3 bands in Figure 5. These findings were confirmed by the core-periphery analysis, which identified a core group of 4 users and a secondary group of 5 other users that accounted for the bulk of traffic on the forum. . This finding is consistent with other research that has found evidence of a core group of users producing the bulk of the content within online communities. The exact size of the core creating the content in varies by application. Some studies have found the core to comprise upwards of 50% of the users [35-37], while other studies have found the numbers to be smaller [38]. Since large networks are expected to contain more lurkers [37], there would be an expected relationship between increasing the size of the network and decreasing the proportion of users in the core. This project identified 13% and 29% in the 2-mode and 1-mode cores respectively, confirming the findings in previous research. The coreness measures in Table 4 range from 0.47 to 0.01, demonstrating that the members that are at the very core of the network (ie, with the highest coreness ratings) are not fully connected to every other member of the network.

It is not a requirement that all community members contribute, and the presence of lurkers within discussion forums is well established [39], but encouraging participation is a key component of the KT process, and it is easier to connect knowledge sharers to knowledge seekers when the knowledge seekers make themselves known. A reasonable number of members were selective posters or lurkers, which from a KT perspective is acceptable, as these members are still participating in the KT exercise by receiving the shared knowledge and applying it to their clinical practices. It is prudent to harmonize the knowledge sharing levels of all members of the community, whereby lurkers are better encouraged and engaged so that they can contribute to the discussions they are reading. By engaging non-contributing knowledge seekers with the knowledge sharers, the ties within the community will strengthen, which in turn will instigate the emergence of discussion topics, validation of existing knowledge and improved flow of knowledge throughout the community. Tools should be developed to allow lurkers to connect with the users contributing knowledge, while active knowledge sharers need mechanisms to inform them when their contributions are used.

It is interesting to note that due to their own interest and activity certain members evolved as KT champions within the community without being explicitly engaged as such by the research team. This organic assumption of leadership and centrality roles, without an official designation or explicit responsibilities, is good for the sustainability of the KT program as it shows that members are engaged and are willing to facilitate the KT exercise because they see the value of the experiential knowledge being shared. From a KT perspective, we believe that it is these individuals who should be engaged as champions or knowledge brokers in future KT programs. Note that these central KT champions need not be experts in their fields, or even necessarily contributing valuable knowledge. Junior members that have a passion for KT and actively use the online discussion forum for knowledge seeking can engage other members of the community to contribute to the discussions, increasing the connectivity of all members within the community. Knowledge seeking activity plays a vital role within the community, as it instigates discussion and encourages communication between members.

Given that a Web 2.0 based discussion forum provides open access to all its members, we investigated whether the online community was exploiting this open communication medium to interact with practitioners from different institutions and professions. At the onset of the project there was a concern that nurses may not be comfortable communicating with physicians (and vice versa) as this was the case in face-to-face practice, but it was encouraging to observe strong interprofessional ties within the discussion forum. This confirms that in an online setting, where there is no face-to-face interaction, professionals felt more comfortable to interact with not just their peers but also with their seniors/juniors. Likewise, we note strong interinstitution ties between members, indicating that regional or institutional preferences were not a factor. From a KT perspective, this finding is particularly relevant as it suggests that an online communication medium is a more open and accessible KT medium for health practitioners, especially for those practitioners who perceive hierarchical and professional classifications as barriers to their knowledge seeking and sharing aspirations.

We would like to point out certain shortcomings of our research. We could not perform any content analysis of the actual online discussion because the discussions were in a foreign language. Our previous research [40] provided a method to process the content of online conversations and to link it to medical keywords. However, due to the language constraint we were unable to apply our method [40], which we believe could have provided a second 2-mode network, linking threads to keywords, thus allowing the analysis of the community in terms of their usage of salient keywords and concepts. In the future, we plan to pursue the translation of the content of the Thai discussion forum, either manually or using automated tools, processing the content of the discussion and then assigning a cloud of medical keywords (based on a medical terminology system, such as MeSH) to the threads.

There is a temporal nature to the data that was not explored in detail. Investigations into response times for new messages, temporal usage patterns for individuals and groups, the time over which an individual thread receives new posts, and the time after the last post that a message receives views are some examples of how time can be incorporated into the analyses. We believe that future investigations into such temporal methods may be able to provide more insights into how users are accessing the system, and in particular to evaluate the culture of collaboration within the LINKS model and how users are integrating the system into their daily work flow.

We believe that more complex SNA methods can be used to evaluate additional aspects of the discussion forum, particularly the use of Exponential Random Graph Models (ERGM) [41,42] can be used to test specific hypotheses about the social network, as well as to incorporate the member and thread attributes into the analysis. ERGM can answer specific hypothesis questions related to the structure of the network, and can connect those hypotheses to components of the LINKS model. In the future, the social network analysis could also be expanded by exploring direct ties within the 1-mode network. The current 1-mode network uses undirected ties to represent 2 users communicating on the same thread, which represents a kind of “friendship”. A directed implementation of the data changes the interpretation of a tie from representing friendship to representing direct communication. Directed networks would present the network in a different light, and would allow the use of other SNA methods, such as prestige centrality [23,24].

The LINKS model was designed as a way to facilitate KT using Web 2.0 tools, but it did not include an evaluation system for the model. This paper has demonstrated how SNA can be used as a tool to evaluate the performance of a discussion forum, but further research is needed. Future work should focus on how SNA methods can be used to directly evaluate the principles of the LINKS model. Once this is done, a set of pre-determined tests can be developed for evaluating systems as they evolve, providing a feedback mechanism for monitoring the health of Web 2.0 tools and ensuring that they are providing the best possible service to their members.

## Multimedia Appendix 1

The 1-mode network, partitioned into the core (top-left) and the periphery. The number in cell [i,j] indicates that those users have communicated on that many threads together. Note that this is a symmetric matrix as the number of messages between users [i,j] is the same as [j,i].

## Multimedia Appendix 2

This is the 2-mode network, partitioned into core (top-left) and periphery. A 1 indicates that a user (columns) communicated on a thread (rows).

## Abbreviations

KT: Knowledge Translation
SNA: Social Network Analysis
LINKS: Leveraging Internet for Knowledge Sharing
ERGM: Exponential Random Graph Models
