# Supplementary material for: Applying Social Network Analysis to Understand the Knowledge Sharing Behaviour of Practitioners in a Clinical Online Discussion Forum
Source: J Med Internet Res. 2012 Dec 4;14(6):e170. doi: 10.2196/jmir.1982 (PMC3799555; doi:10.2196/jmir.1982)
Supplement: Supplementary file 1 [file jmir_v14i6e170_app1.pdf]

	16 U6	2 U1	12 U5	11 U4	20 U6	22 U6	7 U1	23 U6	29 U8	3 U1	10 U3	6 U1	13 U5	4 U1	9 U2	1 U1	17 U6	18 U6	19 U6	5 U1	21 U6	15 U6	8 U2	24 U7	25 U7	26 U7	27 U7	28 U8	14 U5	30 U8	31 U9
U62		10	7	10	8	11	22	13	22	2	2		2	1	2	2	2	4		5	2	2	1	1	2	3	3	1		7	6
U131	10		1	6	4	6	9	3	6	2	1	1		2		1	1	2		4						1	1			4	2
U545	7	1		5	6	7	10	11	8						2			2			1	1	1		2		1	2			4
U473	10	6	5		6	12	13	10	8		1	2	1		1		1	1		1	1	1			1			1		2	4
U646	8	4	6	6		10	6	8	10	1			1	1				2		2	1	2			1			1		1	5
U67	11	6	7	12	10		14	10	8	1		1	1	2	1			3	2	1	2	1	1	1	1	1	1	1		1	10
U173	22	9	10	13	6	14		15	21		4		2		5	1	1	6	1		2	2	1	2	2	4	1	2	2	2	8
U68	13	3	11	10	8	10	15		12		2		1		1			1			1	1	1		1		1	2			4
U87	22	6	8	8	10	8	21	12		1	2	1	1	1	4	1	1	3		3	1	2		1	2	1	2	1		4	7
U132	2	2			1	1			1					1				2		2							1			1	
U305	2	1		1			4	2	2																						2
U137		1		2		1			1																					1	
U57	2			1	1	1	2	1	1															1		2					1
U133	1	2			1	2			1	1										1											
U280	2		2	1		1	5	1	4									1							1						1
U129	2	1					1		1								1			1				2			2			2	
U63	2	1		1			1		1							1				1							1			4	
U64	4	2	2	1	2	3	6	1	3	2					1					1			1		1		2		1	1	1
U642						2	1																								1
U134	5	4		1	2	1			3	2				1		1	1	1						1		1	2			3	
U66	2		1	1	1	2	2	1	1																1						1
U60	2		1	1	2	1	2	1	2																						1
U2	1		1			1	1	1										1									1				1
U72	1					1	2		1				1			2				1						2	3		1	1	
U73	2		2	1	1	1	2	1	2						1			1			1										1
U74	3	1				1	4		1				2							1				2					1	1	
U78	3	1	1			1	1	1	2	1						2	1	2		2			1	3						2	1
U83	1		2	1	1	1	2	2	1																						1
U59							2											1						1		1					
U88	7	4		2	1	1	2		4	1		1				2	4	1		3				1		1	2				
U99	6	2	4	4	5	10	8	4	7		2		1		1			1	1		1	1	1		1		1	1			
