# Supplementary material for: Applying Social Network Analysis to Understand the Knowledge Sharing Behaviour of Practitioners in a Clinical Online Discussion Forum
Source: J Med Internet Res. 2012 Dec 4;14(6):e170. doi: 10.2196/jmir.1982 (PMC3799555; doi:10.2196/jmir.1982)
Supplement: Supplementary file 2 [file jmir_v14i6e170_app2.pdf]

	16U	2U	22U	11U	12U	7U	23U	29U	3U	6U	10U	9U	13U	4U	5U	1U	17U	18U	19U	20U	21U	15U	8U	24U	25U	26U	27U	28U	14U	30U	31U
T100	1		1		1	1	1											1					1				1				1
T101	1					1																				1					
T102						1	1				1																				
T103						1	1																								
T32																1								1			1				
T105	1	1		1	1	1	1																								
T34	1	1													1					1											
T135			1		1																										
T108	1				1	1		1				1						1							1						
T137	1		1	1			1	1												1											
T110					1	1		1				1																			1
T39	1	1		1		1		1																							
T112			1	1	1		1																								
T113			1																	1											
T114			1			1													1												
T43															1											1					
T72																								1							
T145								1																							1
T46	1	1						1							1		1										1			1	
T147	1					1		1												1		1									1
T120		1	1	1		1												1		1											
T121	1	1	1			1	1	1																							
T150					1	1	1																								
T123	1		1					1																							1
T124		1																													
T24	1							1							1	1								1			1			1	
T94	1					1		1			1																				
T127	1					1		1										1													
T98			1			1	1																								
T57	1		1																												1
T130	1			1		1	1	1																							
T131	1		1	1	1	1	1	1												1	1				1						1
T60																								1							
T140					1		1	1												1											
T134			1	1	1	1	1													1								1			1
T62		1				1					1																				1
T136	1		1	1			1	1					1							1											1
T36			1					1						1						1											
T65						1																									
T139	1				1	1	1	1																				1			
T28	1	1														1															
T26																1	1													1	
T142	1		1	1	1	1	1	1												1		1									
T93			1	1		1						1																			
T29	1																									1				1	
T30									1									1									1				
T149						1		1																							
T48	1	1	1					1	1						1			1		1										1	
T148	1							1																							
T23						1							1											1		1					
T58		1				1																									
T81	1			1		1	1	1			1																				
T99			1			1																									
T79						1		1																							
T119						1																									
T133																		1													
T122		1	1			1														1											1
T126						1																									
T141					1		1	1												1											
T146						1		1																							
T143	1				1		1	1												1											
T104							1																								
T33			1																							1					
T31																											1				
T35		1						1																		1					
T109			1	1																											
T138					1																										
T38		1	1												1																
T111			1																1												1

```
 T40 |     1           |   1                                           |
T128 |     1 1         |                                             1 |
 T42 |   1   1         |   1                                       1   |
T116 |         1 1     |                                               |
T117 |     1           |                                               |
 T45 | 1 1             | 1         1 1                                 |
T118 |         1       |                                               |
 T47 | 1     1   1     |                 1                         1   |
 T41 |                 |                 1                         1   |
 T49 | 1         1   1 |                                           1   |
 T21 |           1     |                               1   1     1     |
 T51 |   1   1   1     |                                               |
T125 | 1               |                                               |
 T25 |           1     |               1                               |
 T54 | 1 1             |                                           1   |
 T55 |                 |     1                                       1 |
 T56 |           1   1 |                                             1 |
T115 |     1   1       |                                               |
 T44 |       1         |             1                                 |
 T59 |     1           |                                             1 |
T132 |       1   1   1 |                                               |
 T61 |     1           |                               1               |
T107 |           1   1 |       1                                       |
 T63 |   1           1 |                                               |
 T64 |           1     |                                               |
 T37 |     1           |                                               |
 T66 |           1     |                   1                     1     |
 T67 |                 |                   1                           |
 T68 | 1         1     |         1                         1           |
 T69 |           1     |                                               |
 T70 |           1     |                                               |
 T71 |                 |                               1     1         |
T144 |         1       |                                               |
 T73 |     1     1     |                                               |
 T74 |           1     |                                               |
 T75 |           1     |                                               |
T106 | 1         1 1 1 |       1                                       |
 T80 | 1         1   1 |                                               |
 T50 | 1 1 1 1   1 1   |                                               |
 T82 | 1         1     |                                               |
 T52 |               1 |   1                                           |
 T53 |       1         |   1                                           |
 T95 |     1 1         |                                               |
 T96 |           1     |                   1                           |
 T27 |                 |               1                               |
T129 | 1   1     1     |                         1                     |
      ------------------------------------------------------------------
```
